# Chlorpromazine affects autophagy in association with altered Rag GTPase–mTORC1–TFEB signaling

**DOI:** 10.3389/fcell.2023.1266198

**Published:** 2023-09-08

**Authors:** Ningning Li, Lingling Rao, Xueqing Zhao, Junwen Shen, Dan Su, Guoqiang Ma, Shan Sun, Qilian Ma, Li Zhang, Chunsheng Dong, Kin Yip Tam, Jochen H. M. Prehn, Hongfeng Wang, Zheng Ying

**Affiliations:** ^1^ Jiangsu Key Laboratory of Neuropsychiatric Diseases and College of Pharmaceutical Sciences, Soochow University, Suzhou, China; ^2^ Faculty of Health Sciences, University of Macau, Taipa, China; ^3^ Department of Physiology and Medical Physics and Future-Neuro Research Centre, Royal College of Surgeons in Ireland, Dublin, Ireland; ^4^ Key Laboratory of Nuclear Medicine, Ministry of Health, Jiangsu Key Laboratory of Molecular Nuclear Medicine, Jiangsu Institute of Nuclear Medicine, Wuxi, China; ^5^ Institutes of Biology and Medical Science, Soochow University, Suzhou, China

**Keywords:** CPZ, TFEB, autophagy, mTORC1, Rag GTPases

## Abstract

Autophagy is a critical protein and organelle quality control system, which regulates cellular homeostasis and survival. Growing pieces of evidence suggest that autophagic dysfunction is strongly associated with many human diseases, including neurological diseases and cancer. Among various autophagic regulators, microphthalmia (MiT)/TFE transcription factors, including transcription factor EB (TFEB), have been shown to act as the master regulators of autophagosome and lysosome biogenesis in both physiological and pathological conditions. According to the previous studies, chlorpromazine (CPZ), an FDA-approved antipsychotic drug, affects autophagy in diverse cell lines, but the underlying mechanism remains elusive. In our present study, we find that CPZ treatment induces TFEB nuclear translocation through Rag GTPases, the upstream regulators of mechanistic target of rapamycin complex 1 (mTORC1) signaling. Meanwhile, CPZ treatment also blocks autophagosome–lysosome fusion. Notably, we find a significant accumulation of immature autophagosome vesicles in CPZ-treated cells, which may impede cellular homeostasis due to the dysfunction of the autophagy–lysosome pathway. Interestingly and importantly, our data suggest that the expression of the active form of Rag GTPase heterodimers helps in reducing the accumulation of autophagosomes in CPZ-treated cells, further suggesting a major contribution of the Rag GTPase–mTORC1–TFEB signaling axis in CPZ-induced autophagic impairment.

## Introduction

Autophagy is a critical cellular quality control system used to remove damaged organelles and maintain cellular homeostasis in response to stress. Following stress, intracellular cargos are sequestered by autophagosomes (double-membrane vesicles in the autophagy system) and further delivered to lysosomes for degradation and recycling (Dikic and Elazar, 2018). Based on the specificity of cargos, autophagy can be divided into several selective forms, including mitophagy (autophagy of mitochondria), ER-phagy (autophagy of the endoplasmic reticulum), pexophagy (autophagy of peroxisomes), ribophagy (autophagy of ribosomes), and aggrephagy (autophagy of aggregated proteins) (Stolz et al., 2014). According to the previous studies, more than 30 identified autophagy-related proteins and many important regulators participate in autophagy (Dikic and Elazar, 2018). Autophagy-related gene mutations and autophagic dysfunction are strongly associated with various diseases, including schizophrenia, neurodegenerative diseases, and cancer (Merenlender-Wagner et al., 2013; Schneider et al., 2016; Doherty and Baehrecke, 2018; Vargas et al., 2023).

The microphthalmia (MiT/TFE) transcription factors, including transcription factor EB (TFEB), TFE3, TFEC, and MITF, play important roles in lysosome biogenesis and autophagy. The phosphorylation status of these transcription factors is regulated by multiple kinases and is correlated with intracellular localization and the activity of these transcription factors (Takahara et al., 2020; Morais et al., 2023; Yang et al., 2023). Mechanistic target of rapamycin complex 1 (mTORC1) is one of the major kinases of these transcription factors, and mTORC1 is recruited to the lysosomal surface by the heterodimeric RagA/B–RagC/D GTPases (Saucedo et al., 2003; Sancak et al., 2008). Under normal conditions, the active form of Rag GTPases (GTP-bound RagA/B along with GDP bound RagC/D) associates with these transcription factors, which are instantly phosphorylated by mTORC1 on the surface of lysosomes. Phosphorylated transcription factors interact with YWHA/14-3-3 and remain in the cytosol (Martina and Puertollano, 2013; Xia et al., 2016; Wang et al., 2020). Upon multiple stress stimuli, mTORC1 dissociates from lysosomes and loses its activity. Inactive mTORC1 fails to phosphorylate these transcription factors and, therefore, leads to the nuclear translocation of these transcription factors and the transcriptional activation of lysosomal and autophagosomal genes (Roczniak-Ferguson et al., 2012; Xia et al., 2015; Wang et al., 2020).

Chlorpromazine (CPZ), also called wintermin, is a phenothiazine derivative that is commonly used to treat psychiatric disorders by inhibiting dopamine receptors. Despite CPZ being a classic antipsychotic drug, it exhibits significant adverse drug reactions (side effects) in the clinical applications. In recent years, CPZ has been shown to exhibit different effects on autophagy in various cell lines (Shin et al., 2013; Li et al., 2016; Matteoni et al., 2021; Xu et al., 2022). Several studies have demonstrated that CPZ also exerts anticancer activities by targeting autophagy and other mechanisms (Jhou et al., 2021; Matteoni et al., 2021; Xu et al., 2022). However, the potential mechanism of CPZ-modulating autophagy is still poorly understood. Here, in this study, we find that CPZ displays dual effects on the autophagy flux, suggesting that it not only induces TFEB nuclear translocation in a Rag GTPase-dependent manner (upstream signaling of the autophagy–lysosome pathway, which is associated with autophagosome and lysosome biogenesis) but also impedes the fusion between autophagosomes and lysosomes (downstream signaling of the autophagy–lysosome pathway), thereby overwhelming the overall homeostasis of the autophagy–lysosome system.

## Materials and methods

### Plasmid construction

The following plasmids used in this study, including the empty vectors expressing the tag, namely, mCherry–EGFP–LC3, mCherry–LC3, EGFP–Parkin, mCherry–Parkin, mt–mKeima, pEGFP-N3–TFEB, LAMP1–RFP, pEGFP-N1–TFE3, pEGFP-N1–MITF, pRK5–HA-GST-RagA^GTP^ (RagA^Q66L^), and pRK5–HA-GST-RagC^GDP^ (RagC^S75L^), were described previously (Ying et al., 2009; Ma et al., 2011; Ying et al., 2011; Wang et al., 2012; Tao et al., 2015; Xia et al., 2016; Mao et al., 2017; Chen et al., 2019; Zhang et al., 2020).

### Cell culture, transfection, and chemicals

Human embryonic kidney 293 (HEK 293) cells were cultured in Dulbecco’s modified Eagle’s medium (DMEM; Gibco, 11995500) containing 10% fetal bovine serum (FBS; allBIO, MN220610) with penicillin (100 U/ml) and streptomycin (100 μg/mL). The plasmids were transfected into cells using the Hieff Trans™ Liposomal Transfection Reagent (Yeasen, 40802ES02) in Opti-MEM (OMEM; Gibco, 31985070) without the serum. Furthermore, these cells were then incubated with drugs for the indicated concentrations and time. The following drugs were used: chlorpromazine hydrochloride (MCE, HY-B0407A), aspirin (MCE, HY-14654), ropinirole hydrochloride (MCE, HY-B0623A), chloroquine (CQ; Sigma, C6628), Torin 1 (Tocris Bioscience, 4247) or antimycin A (Sigma, A8674), and oligomycin A (Selleck, S1478). It should be noted that chlorpromazine hydrochloride functions as CPZ, so CPZ refers to chlorpromazine hydrochloride in our present study.

### Immunoblot

The cells were collected and lysed in a cell lysis buffer (50 mM Tris–HCl (pH 7.6) containing protease inhibitor cocktail (Roche, 4693132001), 0.5% sodium deoxycholate, 1% NP-40, and 150 mM NaCl). The proteins were separated by 13% polyacrylamide gel electrophoresis (SDS–PAGE) and then transferred onto a polyvinylidene difluoride membrane (PVDF membrane; Millipore, IPVH00010). The following primary and secondary antibodies were used: anti-GAPDH (Proteintech, 60,004-4-lg; 1:8000), anti-LC3 (Novus Biologicals, NB1-02200; 1:1000), anti-p62 (Enzo Life, BML-PW9860; 1:500), anti-phospho-p70S6K (T389) (Cell Signaling Technology, 9205; 1:100), anti-p70S6K (Epitomics, 1494–1; 1:1000), anti-TFEB (Cell Signaling Technology, 4240; 1:1000), anti-phospho-TFEB (S211) (Cell Signaling Technology, 37,681; 1:100), and RagB antibodies (Santa Cruz Biotechnology, sc-293349; 1:100) over night at 4°C; and horseradish peroxidase conjugated sheep anti-mouse and anti-rabbit antibodies (Jackson ImmunoResearch Laboratories) for 2 h at room temperature. The proteins were visualized using an ECL detection kit (Vazyme, E411-04).

### Immunofluorescence and live cell imaging

HEK 293 cells were fixed with 4% paraformaldehyde for 10 min at room temperature after transfection or treatment and were then permeabilized with 0.1% saponins for another 10 min. The cells were incubated with the anti-HA (Santa Cruz Biotechnology, sc-805; 1:300), anti-LAMP2 (Santa Cruz Biotechnology, sc-18822; 1:250), or anti-mTOR antibody (Cell Signaling Technology, 7C10; 1:500) over night at 4°C, followed by Alexa Fluor 647-conjugated AffiniPure Donkey Anti-Mouse (Yeasen, 33213ES60; 1:400) or Anti-Rabbit IgG (Yeasen, 33113ES60), Alexa Fluor 488-conjugated AffiniPure Donkey Anti-Rabbit IgG (Proteintech, SA00013-2; 1:400), or Alexa Fluor 594-conjugated AffiniPure Donkey Anti-Mouse IgG antibody (Proteintech, SA00013-3; 1:400) for 2 h at room temperature. Hoechst (Sigma, 23,491-45-4) staining was performed for 20 min after fixation to identify the nucleus. The stained cells and live cells were visualized using ZEISS and Nikon confocal microscopes (Wu et al., 2012; Ren et al., 2016; Fang et al., 2017), or a Nikon Ti2-E fluorescence microscope integrated with a pco.edge 4.2 bi sCMOS camera.

### Statistical analysis

ImageJ was used in image processing, fluorescence intensity analysis of mt–mKeima, and immunoblot densitometric analysis. We use ImageJ (Analyze-ROI manager) to choose single positive cells and to measure the fluorescence intensity of red and green channels. The ratios of red/green signals of multiple cells were analyzed to reflect the level of mitophagy. The charts were generated using Prism 7.0 (GraphPad Software). Statistical analyses were performed using Student’s *t*-test. The *p*-values and mean are indicated in figure legends.

## Results

### CPZ treatment results in the accumulation of autophagosomes in cells

**FIGURE 1:**
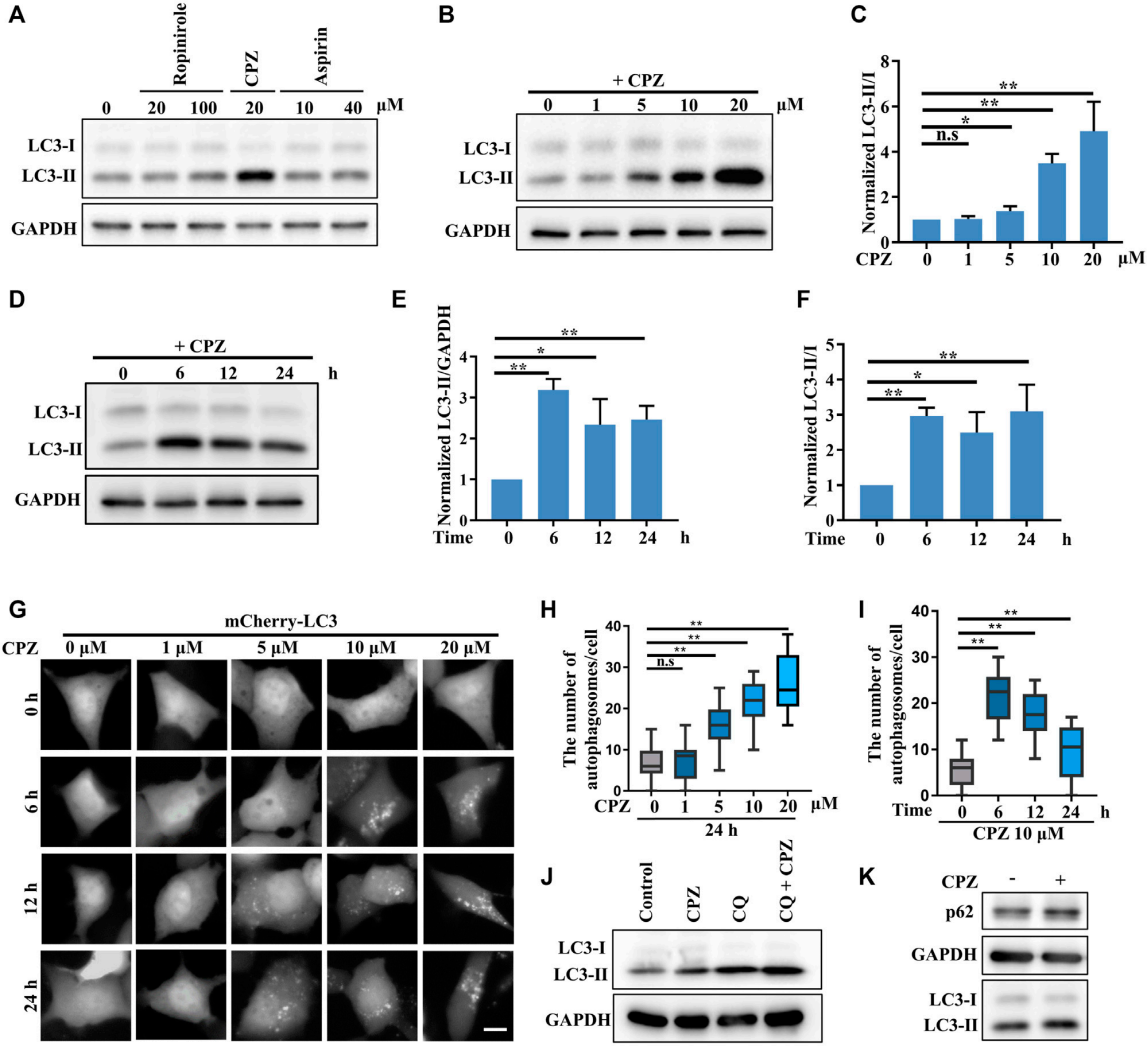
CPZ treatment increases the accumulation of autophagosomes. **(A)** HEK 293 cells were treated with the indicated doses of ropinirole, aspirin, or CPZ for 24 h, and then, the cell lysates were used to detect LC3 levels by immunoblot analysis. **(B)** HEK 293 cells were treated with the indicated doses of CPZ for 24 h, and then, the cell lysates were used to detect LC3 levels by immunoblot analysis. **(C)** Quantification of normalized LC3-Ⅱ/Ⅰ from three independent experiments; mean ± SD; n.s, not significant; *, *p* <0.05; **, *p* <0.01. **(D)** HEK 293 cells were treated with 10 μM CPZ for the indicated times, and then, the cell lysates were used to detect LC3 levels by immunoblot analysis. **(E, F)** Quantification of normalized LC3-Ⅱ/GAPDH or LC3-Ⅱ/Ⅰ from three independent experiments; mean ± SD; *, *p* <0.05; **, *p* <0.01. **(G)** HEK 293 cells were transfected with mCherry–LC3, and then, the cells were treated with indicated doses of CPZ for different times and visualized by fluorescent microscopy. Scale bar, 10 μm. **(H, I)** Quantification of the number of autophagosomes indicated by mCherry–LC3 puncta per cell, given in **(G)**; *n* = 20 cells per sample; mean ± SD; n.s, not significant; **, *p* <0.01. **(J)** A549 cells were treated with 20 μM CPZ or CPZ along with 10 μM CQ for 6 h, and then, the cell lysates were used to detect LC3 levels by immunoblot analysis. **(K)** A549 cells were treated with 20 μM CPZ for 6 h, and then, the cell lysates were used to detect LC3 and p62 levels by immunoblot analysis.

In a primary test of the effects of several FDA-approved central nervous system drugs (including ropinirole, a dopamine agonist that is used to treat Parkinson’s disease; CPZ; and aspirin) on autophagy, we performed a biochemical analysis to examine the level of LC3-II/I, which correlates with the number of autophagosomes, and we unexpectedly found that CPZ treatment strikingly increased the level of LC3-II/I compared with control, ropinirole, or aspirin treatments in HEK 293 cells (Figure 1A). Next, we monitored the turnover of LC3-I to LC3-II upon exposure to different concentrations of CPZ. These data showed that CPZ treatment increased the accumulation of autophagosomes in a dose-dependent manner (Figures 1B,C). Due to severe cytotoxicity with 20 µM CPZ treatment in HEK 293 cells (Supplementary Figure S1), we chose 10 µM CPZ to further examine the turnover of LC3-I to LC3-II over a number of times in HEK 293 cells. We found that the maximum LC3-II accumulation was observed within 6 h after 10 µM CPZ treatment, relative to 12 h or 24 h of CPZ treatment (Figures 1D–F). To further confirm the influence of CPZ on autophagosome formation, we expressed mCherry–LC3 to monitor the number of autophagosomes. Consistent with the aforementioned results, we found that autophagosome accumulation indicated by mCherry–LC3 puncta increased with the concentration of CPZ and the maximum LC3 puncta accumulation was observed within 6 h (Figures 1G–I).

In order to determine if the CPZ-induced accumulation of autophagosomes was due to enhanced autophagosome formation or inhibited autophagosome degradation, we blocked autophagosome degradation with chloroquine, which can intercept autophagy by impairing the autophagosome–lysosome fusion, to further monitor autophagosome formation (Figure 1J). These data showed that CPZ treatment further enhanced the LC3-II level under CQ treatment, indicating CPZ treatment could promote autophagosomal formation by regulating upstream signaling in the autophagy flux. In addition, we measured autophagosome degradation by the protein level of the autophagic substrate p62, and we found that its level was upregulated by CPZ treatment (Figure 1K). Taken together, these data suggest that CPZ strikingly enhances autophagosome accumulation through both enhancing autophagosome formation and blocking the autophagosome–lysosome fusion.

### CPZ has no effect on PINK1/Parkin-mediated mitophagy, a selective form of autophagy

**FIGURE 2:**
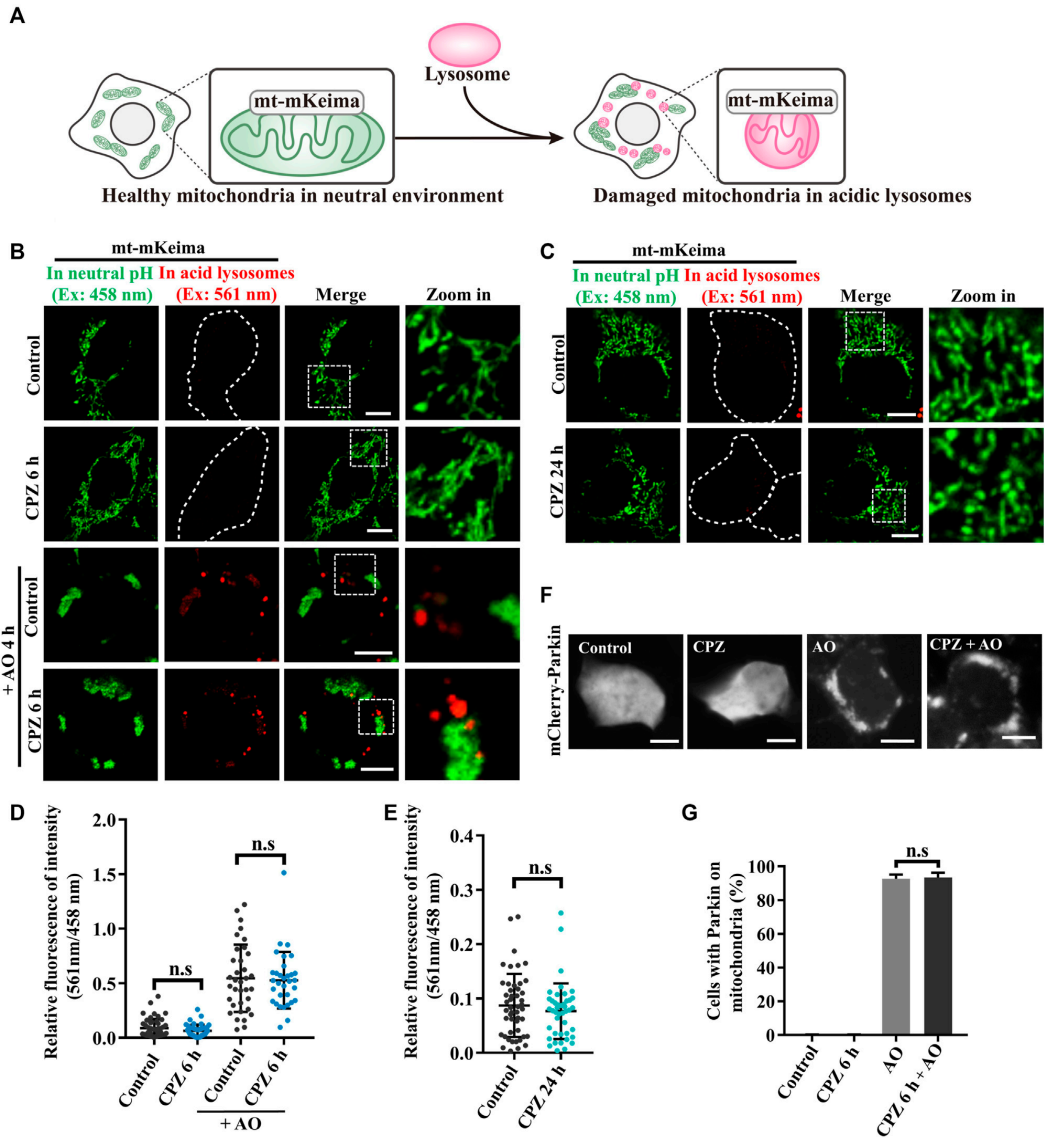
CPZ treatment does not affect PINK1/Parkin-mediated mitophagy. **(A)** Schematic illustration of the mt–mKeima fluorescence reporter for detecting mitophagy. mKeima fusing COX VIII pre-sequence targets to mitochondria. Healthy mitochondria in a neutral environment were observed when green signals were detected from excitation at 458 nm. When damaged mitochondria were delivered to acidic lysosomes, red signals were detected from excitation at 561 nm. **(B)** HEK 293 cells were transfected with mt–mKeima and EGFP–Parkin for 24 h, and then, the cells were treated with 10 μM CPZ for 6 h with or without 5 μg/mL antimycin A and oligomycin A (AO) for 4 h. The cells were visualized by confocal microscopy. Ex, excitation. Scale bar, 10 μm. **(C)** HEK 293 cells were transfected as in **(A)** and were treated with 10 μM CPZ for 24 h. The cells were visualized by confocal microscopy. Ex, excitation. Scale bar, 10 μm. **(D, E)** Quantification of the relative fluorescence intensity of 561 nm/458 nm per mt–mKeima-positive cell after the indicated treatment; n.s, not significant; *n* = 49, 42, 34, and 32 cells in **(D)**; *n* = 48 and 43 cells in **(E)**. **(F)** HEK 293 cells were transfected with mCherry–Parkin for 24 h, and then, the cells were treated with 10 μM CPZ for 6 h, followed by 5 μg/mL AO treatment for 4 h. Scale bar, 10 µm. **(G)** Quantification of the percentage of cells with Parkin recruited on mitochondria from >300 cells; mean ± SD; n.s, not significant.

PINK1/Parkin-mediated mitophagy is a well-known selective autophagy pathway to remove dysfunctional mitochondria and is associated with diverse neurodegenerative diseases (Pickrell and Youle, 2015). Due to the previous studies that show that CPZ decreases the mitochondrial membrane potential and increases reactive oxygen species (ROS), which results in mitophagic initiation (Jhou et al., 2021), we analyzed the effect of CPZ treatment on mitophagy using mt–mKeima, a fluorescence reporter of mitophagy. mt–mKeima localizes in the mitochondrial matrix through its fusion with COX VIII; its excitation peak is approximately 440 nm (green, we use a 458-nm laser for excitation in this study) under neutral conditions but switch to a 586-nm excitation peak (red, we use a 561-nm laser for excitation in this study) under acidic lysosomes (Figure 2A) (Katayama et al., 2011). Our results showed that “561-nm/458-nm” signals were equal in control and CPZ treatment within 6 h or 24 h, indicating that CPZ treatment did not affect the mitophagic flux (Figures 2B–E). In addition, we also had not observed the change in the mitochondrial morphology. In order to determine whether CPZ can affect the mitophagic flux, we first induced mitophagy using antimycin A and oligomycin A (AO, mitochondrial complexes III and V inhibitors) to decrease the mitochondrial membrane potential. The signal of 561 nm/458 nm was significantly increased under AO treatment, which was not affected by CPZ treatment (Figure 2B). The E3 ubiquitin ligase Parkin is one of the key factors in PINK1/Parkin-mediated mitophagy, and it is recruited to damage mitochondria to amplify mitophagy (Kane et al., 2014). Therefore, we also examined Parkin recruitment under CPZ treatment alone or combined with AO treatment. As speculated, CPZ treatment did not affect Parkin localization under normal conditions and AO induction (Figures 2F,G). Taken together, these data suggest that CPZ affects classic macro-autophagy but does not affect the selective form of autophagy, such as PINK1/Parkin-mediated mitophagy.

### CPZ induces TFEB nuclear translocation

**FIGURE 3:**
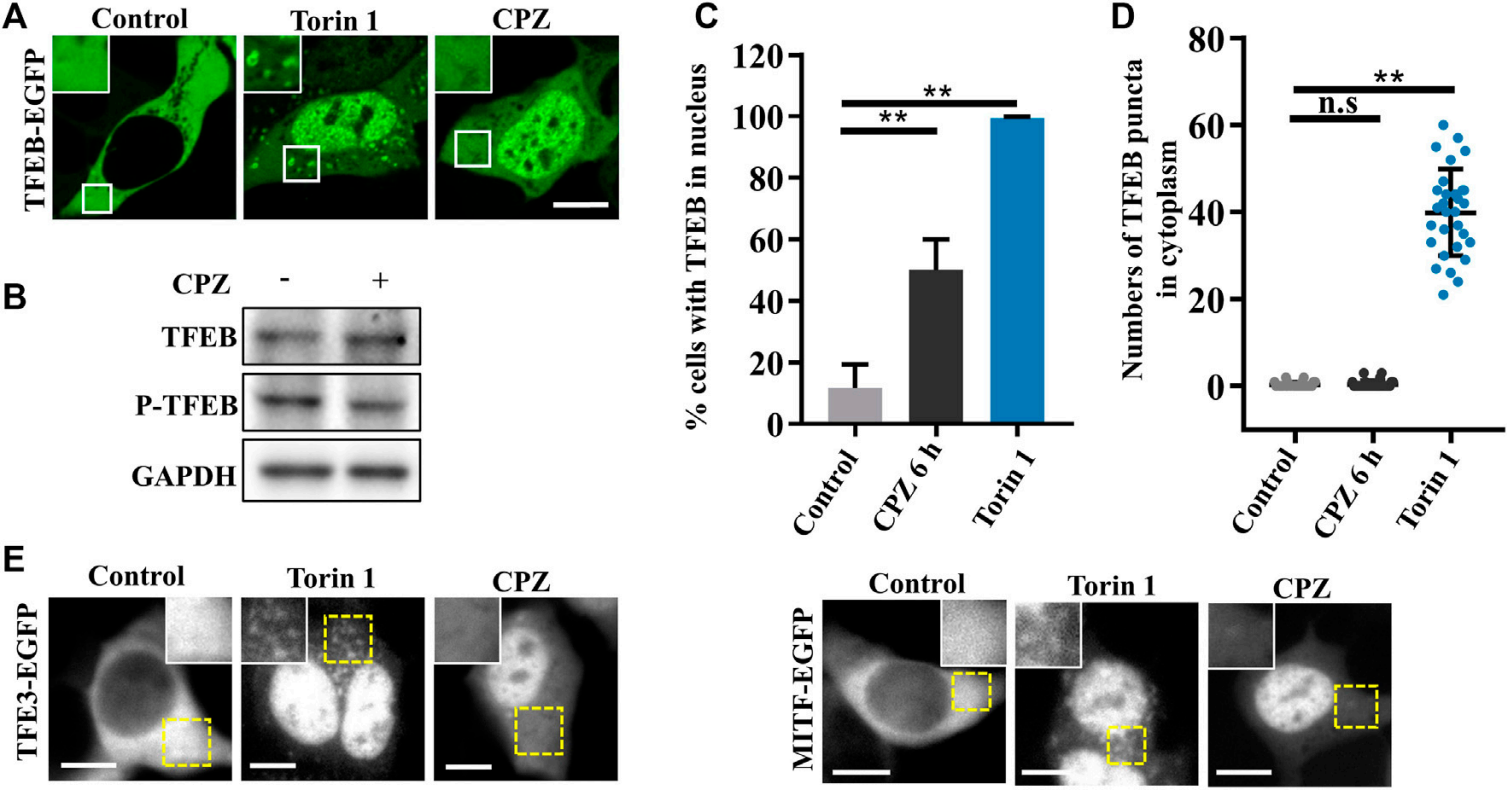
CPZ treatment promotes the nuclear translocation of MiT/TFE transcription factors. **(A)** HEK 293 cells were transfected with EGFP-tagged TFEB and were then treated with 250 nM Torin 1 for 1 h or 10 µM CPZ for 6 h. The cells were visualized by confocal microscopy. Scale bar, 10 µm. **(B)** HEK 293 cells were treated with 10 µM CPZ for 6 h, and then, the cell lysates were subjected to immunoblot analysis using antibodies against TFEB, phospho-TFEB (p-TFEB), and GAPDH. **(C)** Quantification of the percentage of cells in **(A)** with TFEB in the nucleus from ≥300 cells of three independent experiments; mean ± SD; **, *p* <0.01. **(D)** Quantification of the numbers of TFEB puncta in the cytoplasm; *n* = 30 cells per sample, respectively; mean ± SD; n.s, not significant; **, *p *<0.01. **(E)** HEK 293 cells were transfected with EGFP-tagged TFE3 or MITF and were then treated as in **(A)**. The cells were visualized by microscopy. Scale bar, 10 µm.

According to the previous studies, TFEB, one of the most important nucleocytoplasmic shuttling proteins in autophagy signaling, is phosphorylated by mTORC1 and interacts with YWHA/14-3-3 in the cytoplasm under normal conditions. Although it is well-known that TFEB translocates to the nucleus to promote lysosome biogenesis and autophagic activation under stress (Martina et al., 2012; Roczniak-Ferguson et al., 2012), it is worth noting that inactive cytoplasmic TFEB can strongly localize on the lysosomes through its interaction with active Rag GTPases, especially in the case of mTOR inhibition (Martina and Puertollano, 2013; Xia et al., 2016). We wondered whether CPZ enhances autophagosome accumulation by regulating TFEB cellular localization, including lysosomal and nuclear localization. Thus, we used CPZ in treatment for 6 h and Torin 1 (mTORC1 inhibitor) for 1 h in EGFP-tagged TFEB-expressing cells. TFEB is located in the cytoplasm under normal conditions. Compared with this, Torin 1 directly inhibited mTORC1 activity, which resulted in TFEB nuclear translocation and lysosomal localization. However, CPZ treatment only resulted in TFEB nuclear translocation but not lysosomal localization, accompanied by reduced TFEB phosphorylation (Figures 3A–D). Similar effects were observed after CPZ treatment in EGFP-tagged TFE3 or MITF-expressing cells (Figure 3E). Taken together, our results show that CPZ leads to the nuclear translocation of TFEB/TFE3/MITF but not lysosomal localization, suggesting that CPZ can affect the overall Rag GTPase–mTORC1 signaling, but it is unlikely that it affects mTORC1 directly as Torin 1 does.

### CPZ affects TFEB nuclear translocation and mTORC1 activity through Rag GTPases

**FIGURE 4:**
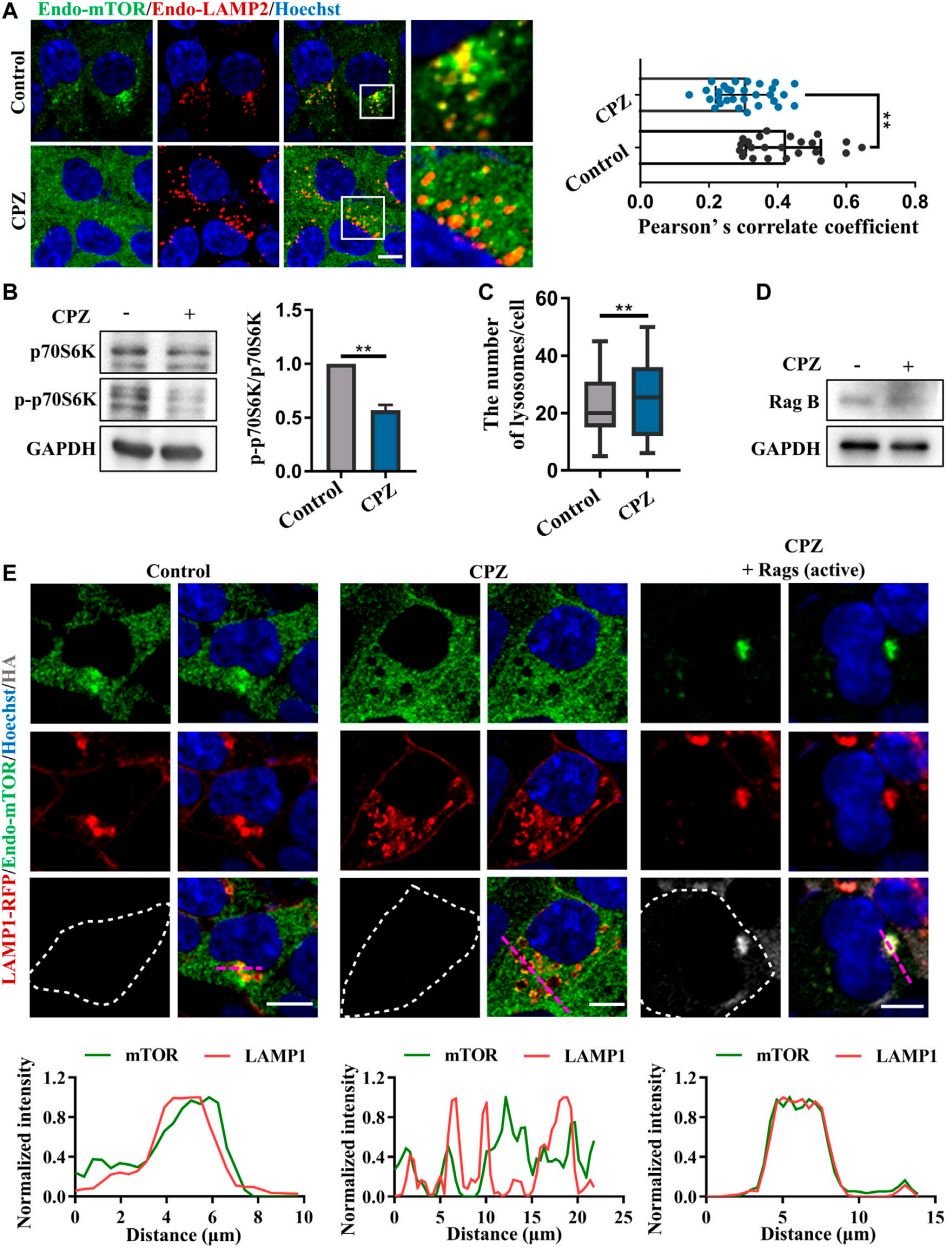
CPZ treatment reduces the lysosome localization of mTORC1 through Rag GTPases. **(A)** HEK 293 cells were treated with CPZ for 6 h, and then, endogenous LAMP2 (red) and mTOR (green) were labeled with corresponding antibodies. Hoechst stain (blue) was used to identify the nucleus. The cells were visualized by confocal microscopy. Scale bar, 10 µm. Co-localization of LAMP2 and mTOR was reflected with Pearson’s correlation coefficient. **(B)** A549 cells were treated with 20 µM CPZ for 6 h, and then, the cell lysates were subjected to immunoblot analysis using antibodies against p70S6K, phospho-p70S6K (p-p70S6K), and GAPDH, and the quantification of normalized p-p70S6K/p70S6K from two independent experiments; mean ± SD; **, *p* <0.01. **(C)** Quantification of the number of lysosomes indicated by LAMP2 puncta in **(A)**; mean ± SD; **, *p* <0.01; *n* = 30 and 30 cells, respectively. **(D)** HEK 293 cells were treated with 10 µM CPZ for 6 h, and then, the cell lysates were subjected to immunoblot analysis using antibodies against RagB and GAPDH. **(E)** HEK 293 cells were transfected with RFP-tagged LAMP1, along with HA-tag or HA-GST-tagged active Rag GTPase mutants (RagA^Q66L^ + RagC^S75L^ = RagA^GTP^ + RagC^GDP^). The cells were treated with 10 µM CPZ for 6 h and stained with anti-mTOR (green) and anti-HA (gray). Hoechst stain (blue) was used to identify the nucleus. The images were obtained by confocal microscopy. Scale bar, 10 µm. The normalized intensity of the corresponding line scan (magenta) was analyzed to show the co-localization of LAMP1 and mTOR.

Given that CPZ induces the nuclear translocation of TFEB/TFE3/MITF, which suggests that mTORC1 fails to phosphorylate these transcription factors, we examined mTORC1 localization and activity, which is associated with TFEB phosphorylation and localization. Our results showed that the lysosomal localization of mTORC1 reduced after CPZ treatment (Figure 4A). Supporting this finding, phosphorylated p70S6K (p-p70S6K) was decreased after CPZ treatment, suggesting that mTORC1 activity is inhibited by CPZ (Figure 4B). Furthermore, we also found that CPZ treatment increased lysosomal numbers, indicating CPZ-induced mTORC1 inhibition promoted TFEB nuclear translocation, followed by lysosomal biogenesis (Figure 4C). Since the CPZ-induced nuclear translocation of TFEB is not caused by the direct inhibition of mTORC1, we wondered whether CPZ treatment inhibited the upstream signaling of mTORC1. Previous studies have shown that the lysosomal localization and activity of mTORC1 are controlled by the Ragulator–Rag GTPase–mTORC1 complex, including the mTORC1 adapter protein raptor, Rag GTPases, and Ragulator (Sancak et al., 2008; Sancak et al., 2010; Zoncu et al., 2011; Bar-Peled et al., 2012). In this complex, active Rag GTPases are pre-required for TFEB lysosomal localization, and active Rag GTPases interact with raptors, which target mTORC1 on the lysosomal surface. So, we first examined the RagB expression, a component of Rag GTPases, and found that it was not affected by CPZ treatment (Figure 4D). The active form of Rag GTPases consists of GTP-bound RagA or RagB and GDP-bound RagC or RagD (Martina and Puertollano, 2013). We next expressed constitutively active Rags (GTP-bound RagA and GDP-bound RagC) and found that they restored the lysosome-like localization of mTORC1 in CPZ-treated cells (Figure 4E).

**FIGURE 5:**
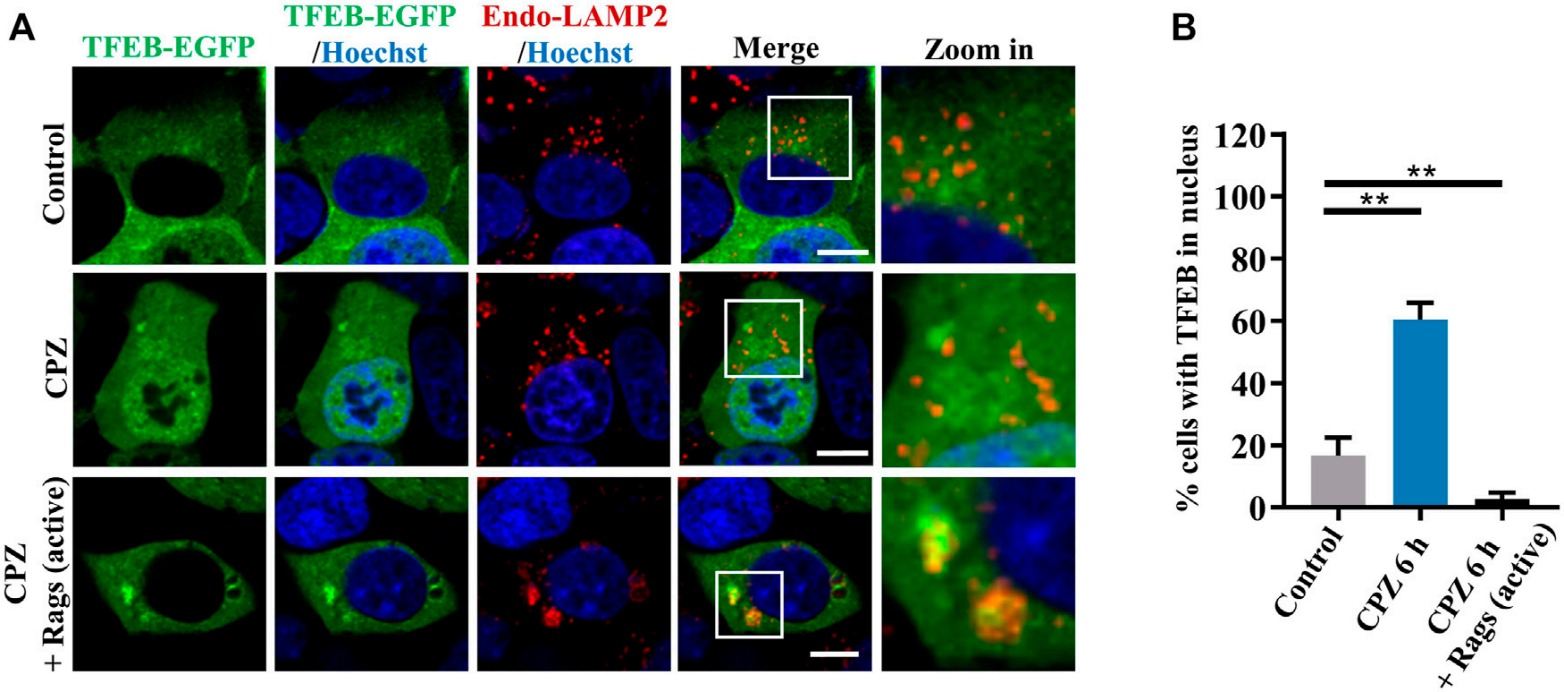
Rag GTPases restore TFEB cellular localization in CPZ-treated cells. **(A)** HEK 293 cells were transfected with EGFP-tagged TFEB, along with HA-tag or HA-GST-tagged active Rag GTPase mutants. The cells were treated with 10 µM CPZ for 6 h and stained with anti-LAMP2 (red). Hoechst stain (blue) was used to indicate the nucleus. The images were obtained by confocal microscopy. Scale bar, 10 µm. **(B)** Quantification of the percentage cells with TFEB in the nucleus from ≥300 cells of three independent experiments; mean ± SD; **, *p* <0.01.

Given that CPZ induces mTORC1 translocation through Rag GTPases, we observe upstream regulators in Rag GTPase–mTORC1–TFEB signaling (Martina and Puertollano, 2013). We further examined the role of Rag GTPases in CPZ-induced TFEB nuclear translocation. Consistent with our previous studies, TFEB was located in the cytoplasm under normal conditions. TFEB shuttled to the nucleus under CPZ treatment, but TFEB nuclear translocation was inhibited by active Rag GTPases, accompanied by the lysosomal localization of TFEB (Figures 5A,B; Supplementary Figures S2A, B). Taken together, our data suggest that Rag GTPases contribute to CPZ-impaired mTORC1 localization and TFEB nuclear translocation.

### Active Rag GTPases reduce the CPZ-induced accumulation of autophagosomes

**FIGURE 6:**
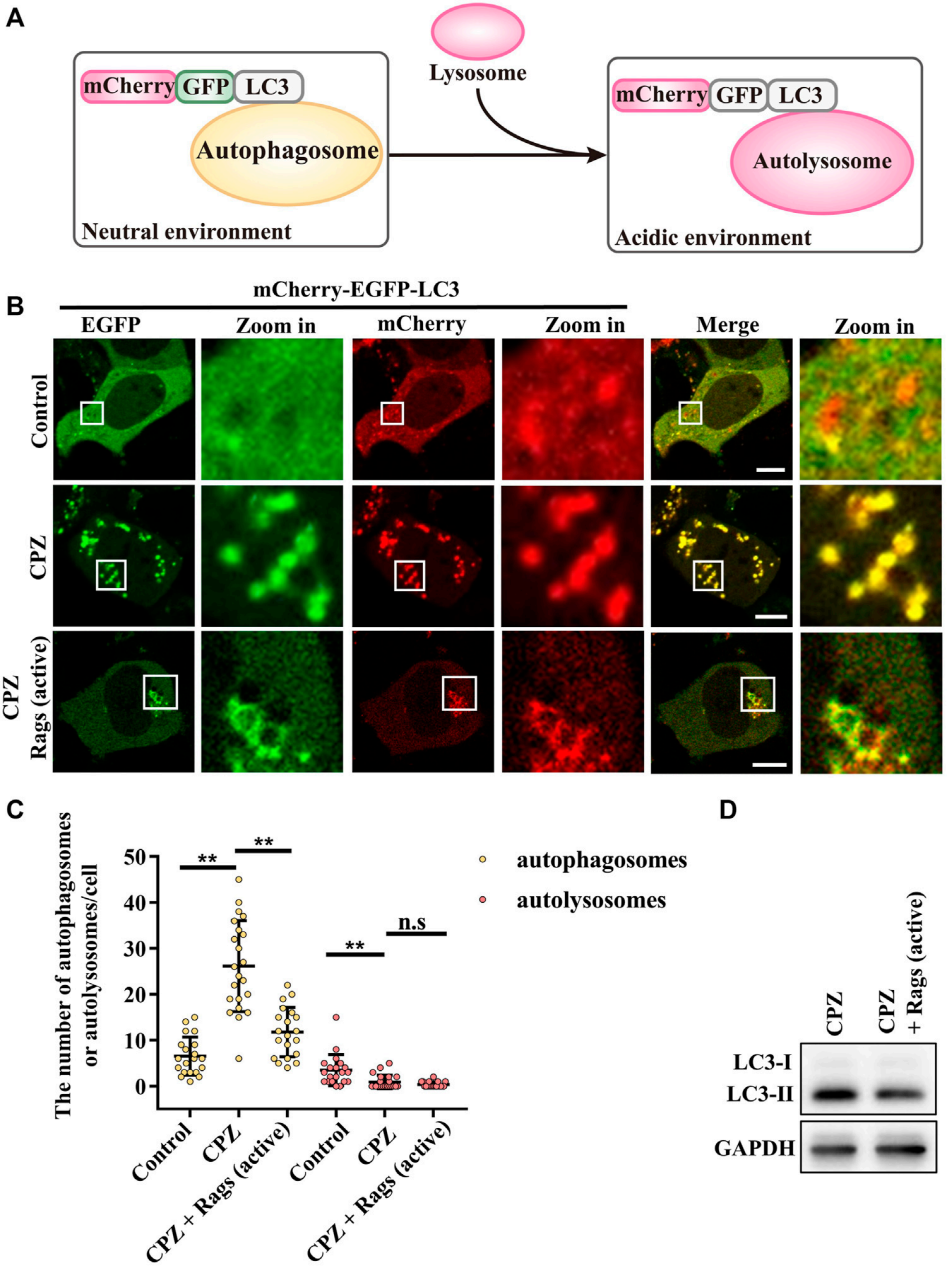
CPZ treatment perturbs the autophagic flux, which can be recovered by the expression of active Rag GTPases. **(A)** Schematic illustration of the mCherry–EGFP–LC3 probe for monitoring the autophagic flux. Under a neutral environment, mCherry signals and green signals co-exist to show yellow puncta, which represent autophagosomes. When autophagosomes are fused with lysosomes, through green fluorescence quenching, mCherry signals alone represent autolysosomes. **(B)** HEK 293 cells were transfected with mCherry–EGFP–LC3, along with HA-tag or HA-GST-tagged active Rag GTPase mutants. Then, the cells were treated with 10 µM CPZ for 6 h. The live cells were visualized by confocal microscopy. Scale bar, 10 µm. **(C)** Quantification of the numbers of autophagosomes (yellow dots) and autolysosomes (red dots); mean ± SD; n.s, not significant; **, *p* <0.01; *n* = 20, 22, 20, 20, 22, and 20 cells, respectively. **(D)** HEK 293 cells were transfected with HA-tag or HA-GST-tagged active Rag GTPase mutants, followed by 10 µM CPZ for 6 h. The cell lysates were used to detect LC3 levels by immunoblot analysis.

To verify the role of Rag GTPases in CPZ-affected autophagy, we used mCherry–EGFP–LC3 to examine the autophagic flux. When autophagosome formation takes place, mCherry–EGFP–LC3 shows yellow dots (red dots overlap with green dots), whereas when an autophagosome fuses with the lysosome, through green fluorescence quenching, mCherry–EGFP–LC3 shows red dots (Figure 6A) (Kuma et al., 2017). Our result showed that CPZ treatment increased the number of yellow dots but decreased the number of red dots, suggesting that CPZ reduces the overall autophagic flux. Interestingly and importantly, the active Rag GTPase expression reduced the accumulation of autophagosomes in CPZ-treated cells (Figures 6B–D). Taken together, our results suggest that CPZ induces excessive autophagosome accumulation, which is alleviated by the active Rag GTPase overexpression.

## Discussion

Interestingly, mitophagy, a selective form of autophagy, which eliminates dysfunctional mitochondria to maintain cellular homeostasis and plays important roles in the aging and neurodegenerative diseases (Rodolfo et al., 2018), was not affected by CPZ treatment (Figure 2). It is observed that Parkin is one of the key factors in the mitophagic progress (Pickrell and Youle, 2015) and is phosphorylated by PINK1, which localizes on the surface of damaged mitochondria. Mitochondria-localized PINK1 leads to the mitochondrial translocation and activation of Parkin as an E3 ubiquitin ligase (Durcan and Fon, 2015); we examined Parkin mitochondrial translocation and found that it did not change in CPZ-treated cells, compared with control cells upon mitochondrial damage (Figures 2F,G). Therefore, we speculate that the OMM proteins are further ubiquitinated by activated Parkin (Narendra and Youle, 2011) and recruit mitophagy receptors, including the amyotrophic lateral sclerosis (ALS)-associated protein optineurin (OPTN), NDP52, and TAX1BP1 (Moore and Holzbaur, 2016; Ryan and Tumbarello, 2018), to a similar extent in control or CPZ-treated cells. Intriguingly, since these receptors can connect with autophagosome proteins, such as ATG8s, to induce the formation of autophagosomes (Nguyen et al., 2016; Kriegenburg et al., 2018), eventually, these autophagosomes will fuse with lysosomes to degrade mitochondria (Lőrincz and Juhász, 2020). CPZ-influenced autophagosomes did not affect the autophagic degradation of damaged mitochondria in our observations (Figures 2B–E). These data suggest that the working mechanism underlying selective autophagy and macro-autophagy may vary. Relevant to this, the Parkin-driven autophagosomal recognition of damaged mitochondria may be both sufficient and redundant for degradation, even if the cells are treated with CPZ. In addition, in this case, the lysosomes may also be overwhelmed due to a huge incoming flux of autophagosomes containing damaged mitochondria, similar to the situation with CPZ-treated cells.

According to the previous studies, active Rag GTPases are required for TFEB-Rag GTPase interactions on lysosome surfaces, but mTORC1 activity is required for further TFEB phosphorylation and nuclear translocation. Therefore, there are two cases of TFEB localization when mTORC1 is inhibited: 1) the active form of Rag GTPases which retain TFEB on lysosomes (despite the major portion of TFEB being translocated into the nucleus upon mTORC1 inhibition) and 2) the inactive form of Rag GTPases which fail to retain TFEB on lysosomes. We found that CPZ resulted in “case (2).” In this case, TFEB has no lysosomal localization since Rag GTPases are affected by CPZ, which is different from “case (1).” Torin 1 treatment will simply inhibit mTORC1 activity but not Rag GTPases (Figures 3A,E) (Martina and Puertollano, 2013).

**FIGURE 7:**
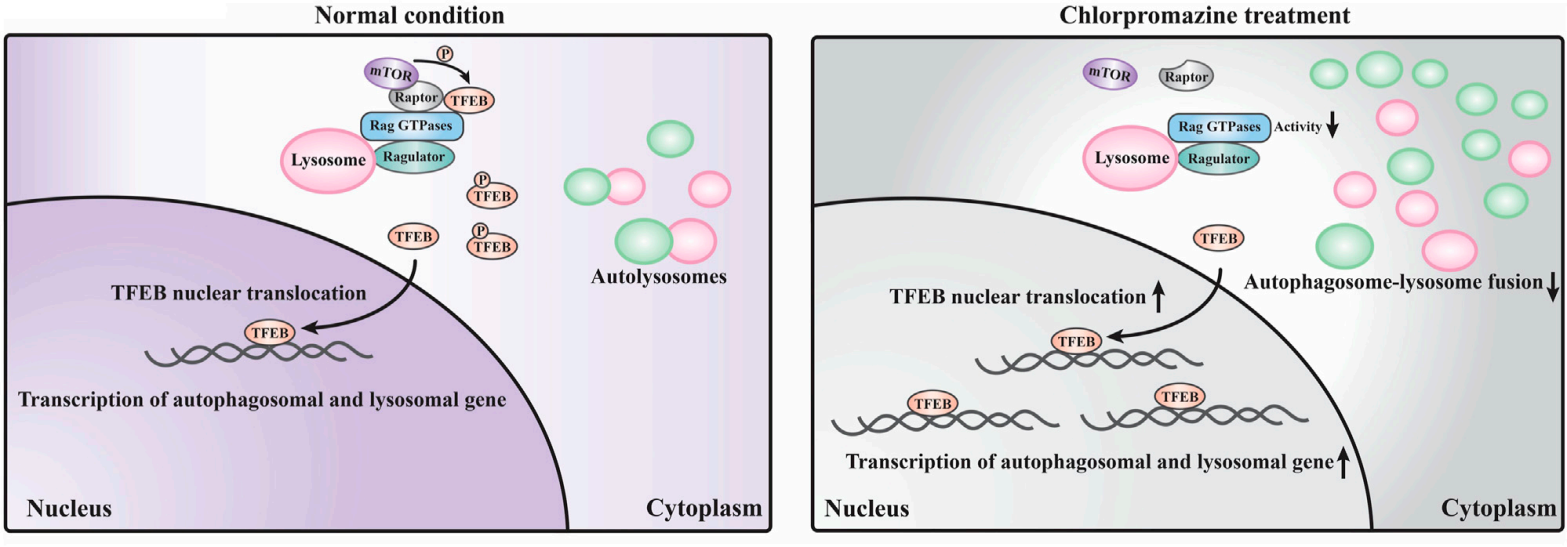
Schematic model of the current study. Under normal conditions, a large portion of TFEB is continuously phosphorylated by mTORC1 (which contains mTOR and raptor) localized on the lysosome surface and remains in the cytoplasm. Only a small portion of TFEB translocates into the nucleus to promote the transcription of autophagosomal and lysosomal genes. Under CPZ treatment, a large portion of TFEB translocates into the nucleus to promote autophagosomal and lysosomal gene transcription due to the decreased activities of Rag GTPases and reduced lysosomal localization of mTORC1. In addition, CPZ treatment also blocks the autophagosome–lysosome fusion, which results in the striking accumulation of immature autophagosomes and lysosomes in the cell.

Our data suggest that the perturbed autophagosome–lysosome fusion and altered TFEB-mediated autophagy may both contribute to CPZ-induced side effects (Figure 7). Impairment of autophagy, especially the impairment of lysosomes, has been reported to regulate mTOR and TFEB localization (Fedele and Proud, 2020). This regulatory progress can be considered a protective response of autophagic impairment, suggesting a key contribution of mTORC1–TFEB signaling to cellular quality control. Although, interestingly, whether Rag GTPases are affected by autophagy or lysosome impairment remains unclear. The evidence that CPZ affects TFEB localization through Rag GTPases prompts us to speculate that CPZ plays two roles in the regulation of autophagy (Figures 5, 6), similar to our previous study which shows that autophagy dysfunction can be induced by dual effects in a neurodegenerative disease model (Xia et al., 2016; Ying et al., 2016). According to that study, the deficiency of TDP-43, a hallmark protein broadly involved in diverse neurological disorders, can lead to neurotoxicity through the enhancement of TFEB-mediated biogenesis of autophagosomes and lysosomes, and the blocking of the autophagosomal and lysosomal fusion (Xia et al., 2016). In summary, we speculate that impaired autophagy may contribute to the clinical adverse drug reaction of CPZ, and the strategy to reduce autophagosome biogenesis or enhance the fusion between autophagosomes and lysosomes, in this case, will be worth exploring.

## Data Availability

The original contributions presented in the study are included in the article/Supplementary Material; further inquiries can be directed to the corresponding authors.
