# Supplementary material for: Chlorpromazine affects autophagy in association with altered Rag GTPase–mTORC1–TFEB signaling
Source: Front Cell Dev Biol. 2023 Sep 8;11:1266198. doi: 10.3389/fcell.2023.1266198 (PMC10514517; doi:10.3389/fcell.2023.1266198)

**Supplementary Material**

# Chlorpromazine harms autophagy in association with altered Rag GTPase-mTORC1-TFEB signaling

**Ningning Li[1], Lingling Rao[1], Xueqing Zhao[1], Junwen Shen[1], Dan Su[1], Guoqiang Ma[1], Shan Sun[1, 2], Qilian Ma[1, 3], Li Zhang[4], Chunsheng Dong[5], Kin Yip Tam[2], Jochen H. M. Prehn[3], Hongfeng Wang[1, *], Zheng Ying[1, *]**

[1]Jiangsu Key Laboratory of Neuropsychiatric Diseases and College of Pharmaceutical Sciences, Soochow University, Suzhou, Jiangsu 215123, China
[2]Faculty of Health Sciences, University of Macau, Taipa, Macau, China
[3]Dept. of Physiology & Medical Physics and FUTURE-NEURO Research Centre, Royal College of Surgeons in Ireland, Dublin 2, Ireland
[4]Key Laboratory of Nuclear Medicine, Ministry of Health, Jiangsu Key Laboratory of Molecular Nuclear Medicine, Jiangsu Institute of Nuclear Medicine, Wuxi, Jiangsu 214063, China
[5]Insititutes of Biology and Medical Science, Soochow University, Suzhou, Jiangsu 215123, China

*Address correspondence to:
Zheng Ying, Jiangsu Key Laboratory of Neuropsychiatric Diseases and College of Pharmaceutical Sciences, Soochow University, Suzhou, Jiangsu 215123, China. E-mail addresses: zheng.ying@suda.edu.cn
Hongfeng Wang, Jiangsu Key Laboratory of Neuropsychiatric Diseases and College of Pharmaceutical Sciences, Soochow University, Suzhou, Jiangsu 215123, China. E-mail addresses: wanghongfeng@suda.edu.cn

**Supplementary Material**

**FIGURE S1. Related to FIGURE 1.**

HEK 293 cells were treated with indicated doses of CPZ for 24 h and followed with propidium iodide (PI) and Hoechst for 20 min. The cell death was qualified of live cells from three independent experiments, means ± SD, n.s, not significant, *, $P < 0.05$, **, $P < 0.01$.

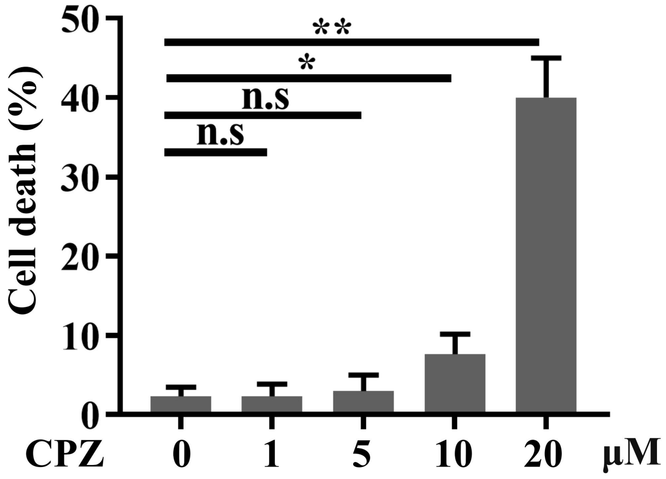

**FIGURE S2. Related to FIGURE 5.**

**(A)** HEK 293 cells were transfected with EGFP-tagged TFEB and HA-GST-tagged active Rag GTPase mutants. The cells were treated with 10 μM CPZ for 6 h and stained with anti-LAMP2 (red) and HA (grey). Hoechst (blue) was used to indicate nucleus. The images were obtained by confocal microscopy. Scale bars, 10 μm. **(B)** Quantification of the percentage cells with TEFB in nucleus from ≥ 300 cells, means ± SD, n.s, not significant.

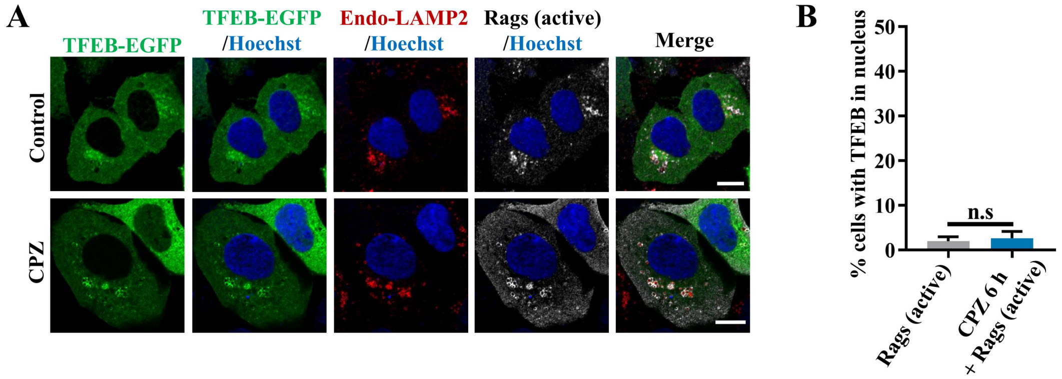

Supplement: Supplementary file 1 [file Table1.pdf]
